# Community assembly and network structure of epiphytic and endophytic phyllosphere fungi in a subtropical mangrove ecosystem

**DOI:** 10.3389/fmicb.2023.1147285

**Published:** 2023-03-17

**Authors:** Chunchao Zhu, Yumiao Lin, Zihui Wang, Wenqi Luo, Yonghua Zhang, Chengjin Chu

**Affiliations:** ^1^Department of Bioengineering, Zhuhai Campus of Zunyi Medical University, Zhuhai, China; ^2^Goldpac Limited, Zhuhai, China; ^3^Département des Sciences Biologiques, Université du Québec à Montréal (UQAM), Montreal, QC, Canada; ^4^State Key Laboratory of Biocontrol, School of Ecology, Sun Yat-sen University, Guangzhou, China; ^5^College of Life and Environmental Science, Wenzhou University, Wenzhou, China

**Keywords:** plant-fungus interaction, fungal network, phylogenetic constraint, true mangroves, mangrove associates, network structure, internal transcribed spacer 2

## Abstract

Microorganisms can influence plant growth and health, ecosystem functioning, and stability. Community and network structures of mangrove phyllosphere fungi have rarely been studied although mangroves have very important ecological and economical values. Here, we used high throughput sequencing of the internal transcribed spacer 2 (ITS2) to assess epiphytic and endophytic phyllosphere fungal communities of six true mangrove species and five mangrove associates. Totally, we obtained 1,391 fungal operational taxonomic units (OTUs), including 596 specific epiphytic fungi, 600 specific endophytic fungi, and 195 shared fungi. The richness and community composition differed significantly for epiphytes and endophytes. Phylogeny of the host plant had a significant constraint on epiphytes but not endophytes. Network analyses showed that plant–epiphyte and plant–endophyte networks exhibited strong specialization and modularity but low connectance and anti-nestedness. Compared to plant–endophyte network, plant–epiphyte network showed stronger specialization, modularity, and robustness but lower connectance and anti-nestedness. These differences in community and network structures of epiphytes and endophytes may be caused by spatial niche partitioning, indicating their underlying ecological and environmental drivers are inconsistent. We highlight the important role of plant phylogeny in the assembly of epiphytic but not endophytic fungal communities in mangrove ecosystems.

## Introduction

Phyllosphere represents the aboveground parts of plants, comprising mainly stems and leaves, which is inhabited by hyperdiverse microbial communities (Lindow and Brandl, 2003; Whipps et al., 2008). Phyllosphere microorganisms include endophytes that live within plant tissues and epiphytes that live on the surface of plant tissues (Lindow and Brandl, 2003). Epiphytes are exposed to many external environmental stressors (e.g., temperature and humidity) and selective pressures that host plants exerted *via* leaf nutrients, morphological and physiological traits (Vacher et al., 2016). In comparison, endophytes live in a more sheltered environment but are selected by nutrients and defence compounds of host plants (Mercado-Blanco, 2015). These two types of microorganisms can influence plant growth and health, and productivity of ecosystems. For instance, epiphytes can reduce disease symptoms in the plant caused by pathogens (Widmer and Dodge, 2013) and endophytes can limit pathogen damage (Arnold et al., 2003) and enhance plant tolerance to abiotic stress (Hubbard et al., 2014). In turn, host plants can affect microbial community composition (Kembel and Mueller, 2014; Ricks and Koide, 2019; Yao et al., 2020) and diversity (Griffin et al., 2019). Thus, elucidating the interactions between host plants and epiphytic and endophytic microorganisms in the phyllosphere can help understand the mechanisms underlying phyllosphere microbial community assembly.

Microbial communities inhabiting mangroves have received increasing attention because mangroves have important ecological roles and provide a wide range of services in environment and economy (Taylor et al., 2003). In mangrove forests, different microbial communities in the phyllosphere of true mangroves and mangrove associates (two categories of mangrove species) are anticipated due to that they have significantly different leaf physiological and ecological traits (Wang et al., 2011). However, only community structure and network properties of phyllosphere fungi on true mangroves have been investigated and reported (Yao et al., 2019). A lack of knowledge about community and network structure of phyllosphere fungi living on mangrove associates may hinder both understanding and generalizations of fungal community assembly rules for mangrove ecosystem.

Community structure between the epiphytic and endophytic phyllosphere fungi has been studied and found to be significantly different in true mangroves and other woody plants (Osono, 2017; Gomes et al., 2018; Yao et al., 2019). Such difference in community structure of epiphytic and endophytic fungi in the phyllosphere can be significantly affected by host plant taxonomy or identity (Kembel and Mueller, 2014; Ricks and Koide, 2019; Yao et al., 2020). Host plant taxonomy and leaf physicochemical properties might influence community composition and structure of phyllosphere endophytic and epiphytic fungi *via* mediating immigration, survival and growth of microbial colonists.

Due to phylogenetic conservatism of functional traits among host plants, plant phylogeny has been shown to play an crucial role in determining the community structure of phyllosphere endophytic fungi (Ricks and Koide, 2019; Darcy et al., 2020) and foliar epiphytic fungal microbiome (Zhu et al., 2021). However, it is still unknown how plant phylogenetic history affects phyllosphere epiphytic and endophytic fungal communities in mangrove forests.

To comprehensively understand plant–microbe interaction patterns and assembly rules of microbial communities, network approaches have been applied to describe the network structures of plant–microbe (Yao et al., 2019, 2020; Zhu et al., 2020, 2022). Two main network properties include nestedness and modularity. The nestedness describes a pattern of interaction where the more specialist species (having few interactions) tend to interact with a subset of the interaction partners of the more generalist species (having many interactions) (Bascompte et al., 2003). Nestedness networks are more robust to species extinctions due to that extinction of specialist species may have little effect on network structure (Memmott et al., 2004; Burgos et al., 2007). Modularity measures the extent to which the network is subdivided into subgroups of species (modules), where species within modules interact more frequently with each other than species between modules (Olesen et al., 2007). Modularity can enhance the stability of networks by limiting perturbations in a module, and buffering the propagation of secondary extinctions throughout the community following disturbance (Stouffer and Bascompte, 2011). Thus, network structures may promote the robustness of ecological networks to species extinction arising from perturbation.

Significantly specialized but a lack of nestedness architecture are exhibited by most plant–root fungus networks (Toju et al., 2015, 2016, 2018). Moreover, highly specialized and modular but less nested structures have been currently reported in leaf epiphytic and endophytic fungal networks (Chagnon et al., 2016; Yao et al., 2019). However, to our knowledge, little is reported about the link between the architectures of mangrove leaf epiphytic and endophytic fungal networks and community stability.

To fully understand fungal community assembly in the phyllosphere of mangroves, we investigated and compared phyllosphere epiphytic and endophytic fungal communities associated with mangroves (including true mangroves and mangrove associates) in South China. First, we hypothesized that species richness and community composition of epiphytic and endophytic fungi differed due to distinct microenvironments provided by leaf tissues and leaf surfaces. Second, we expected to observe different topological structures between epiphytic and endophytic fungal networks. Specifically, the difference in network topological structures of phyllosphere epiphytic and endophytic fungi was expected to lead to different network robustness. Third, we hypothesized that phylogeny of mangrove plants had a stronger constraint on endophytic than epiphytic fungal communities due to that endophytes may be majorly selected by the plant functional traits, whereas epiphytes may be majorly affected by external abiotic factors. The results of this work allow us to fully understand the phyllosphere fungal community assembly in mangrove ecosystems and help to make strategies in conservation and restoration of mangrove forests.

## Materials and methods

### Study site and field sampling

The study was conducted in the Qi’ao Island Mangrove Nature Reserve (113°36′40″–113°39′15″ E, 22°23′40″– 22°27′38″ N), with total area of 5093.0 ha. It is located at Zhuhai city, Guandong province, in South China. This study site belongs to a subtropical monsoon zone. The annual average temperature is 22.4°C, and the annual average precipitation is 1700–2,300 mm. We investigated phyllosphere fungi on six true mangrove species, including *Bruguiera gymnorrhiza*, *Kandelia candel*, *Excoecaria agallocha*, *Acanthus ilicifolius*, *Sonneratia apetala*, *Conocarpus erectus* and five mangrove associate species, including *Hibiscus tiliaceus*, *Cerbera manghas*, *Pluchea indica*, *Thespesia populnea*, *Heritiera littoralis* at the same site. On May 3–5, 2022, we collected leaves from 10 individuals of each of the 11 plant species. The distance among individuals of the same plant species was more than 20 meters. Ten fully expanded and mature leaves with no visible signs of damage or disease were randomly collected from each plant. Leave samples were immediately put in sterile plastic bags, labeled, and subsequently stored at –20°C refrigerator in the laboratory.

### Molecular analysis

We used 5.0 g pooled leaf samples from 10 individuals of each plant species (0.5 g leaf strips per plant individual) to extract the genomic DNA of endophytes and epiphytes, respectively. To obtain epiphytic fungi from leaf surfaces according to Gourion et al. (2006), we first put 5.0 g leaves in a 50-mL EP tube. Then, sterile cooled TE buffer (10 mM Tris–HCl, 1 mM EDTA, pH 7.5) was added to the tube. To mix completely, the tube was subjected to vortexing (30 s) and alternating sonication (45 s) three times. The treated leaves were removed from the tube and the suspension was centrifuged at 10,000 × g for 10 min. We discarded the supernatant and retained the pellet to extract genomic DNA of epiphytic fungi. For endophytic fungi, 5.0 g leaves of each plant species were surface sterilized in 75% ethanol (1 min), 3.25% sodium hypochlorite (3 min), and 75% ethanol (30 s), followed by distilled water for three rinses (Guo et al., 2000). Treated leaves were frozen in liquid nitrogen and ground using a sterilized mortar and pestle. The grind leaves were used to extract genomic DNA of endophytic fungi. We extracted the genomic DNA of epiphytes and endophytes with the Soil DNA Kit D5625 (Omega Bio-tek, America). The concentration of DNA was determined by the NanoDrop 1,000 Spectrophotomter. A two-step PCR procedure was conducted to amplify the ribosomal internal transcribed spacer 2 (ITS2) of phyllosphere fungi. The first PCR amplification of the ITS region was conducted using primers ITS1F (Gardes and Bruns, 1993) and ITS4 (White et al., 1990) in a 50 μl reaction consisting of 1uL forward and reverse primers (10 μM), 25 uL Takara Taq DNA polymerase mixture, 50 ng of template DNA, 1 uL Bovine serum albumin (BSA), and 25–28 μl ddH_2_0. The PCR conditions were 94°C for 5 min, followed by 30 cycles of denaturating at 94°C for 30 s, annealing at 53°C for 30 s, elongation at 72°C for 30 s, and a final elongation at 72°C for 8 min. The second PCR amplification were conducted with the primers fITS7 (Cruz-martinez et al., 2012) and ITS4 (White et al., 1990). Accordingly, a 50 μl reaction consisted of 2 uL forward and reverse primers (10 μM), 25 uL Takara Taq DNA polymerase mixture, 4 uL template (i.e., the first PCR products), and 17 μl ddH_2_0. The PCR conditions were 94°C for 3 min, followed by 30 cycles of denaturation at 94°C for 20 s, annealing at 53°C for 20 s, elongation at 72°C for 30 s, and a final elongation at 72°C for 5 min. All PCR products were visualized using gel electrophoresis. High throughput sequencing was conducted on an Illumina Novaseq PE 250 platform with 2 × 250 base pairs paired-end reading at Magigen company, China.

### Bioinformatics analysis

In Quantitative Insights into Microbial Ecology (QIIME) version 1.7.0 (Caporaso et al., 2010), raw sequences were denoised using DADA2 (Callahan et al., 2016) to keep high-quality sequences. These denoised sequences had an average quality >25 bases and did not include ambiguous base calls, primers and barcode sequences. To assign taxonomic information to fungi, the representative OTU sequences obtained from DADA2 were searched and blasted against the ITS fungal taxonomic classifier training from UNITE v. 6.2 reference database (Abarenkov et al., 2010). To eliminate the differences in the number of reads across samples, we rarefied number of reads per sample to the minimum sequencing depth (78, 264 clean reads). We discarded any sequence which were assigned to non-fungal Eukarya. In addition, to correct the sequencing and PCR errors, we removed the OTUs with less than 10 reads from each sample.

### Statistical analyses

The fungal OTU richness was calculated as the OTU number of fungi observed in a sample. To test whether epiphytic fungi had higher richness than endophytic fungi, paired t-test was carried out after log transformation to make data follow the normal distribution. We constructed a phylogenetic tree of host plant species (Supplementary Figure S1) based on the *rbcL*a fragment downloaded from NCBI database (605 bp, see deposited information in Supplementary Table S1) using maximum likelihood method and Kimura 2-parameter model in Mega software (Kumar et al., 2016). The phylogeny of host plants was used to detect phylogenetic signals in the richness for epiphytic and endophytic fungi on six true mangroves and five mangrove associates according to Blomberg’s *K* statistics (Blomberg et al., 2003) and Pagel’s *lambda* statistics (Pagel, 1999). Based on OTU read data of Hellinger-transformation, we utilized Bray–Curtis method to calculate the dissimilarities of community composition of epiphytic and endophytic fungi. To understand the community composition of epiphytic and endophytic fungi, we conducted non-metric multidimensional scaling (NMDS) analysis in R-package “vegan” (Oksanen et al., 2019). Subsequently, analysis of similarities (ANOSIM) was performed to examine the significance of differences in epiphytic and endophytic fungal communities. ANOSIM gave an R value (the degree of differences between groups) and a *p* value (significant level). To test whether community similarities of epiphytic and endophytic fungi decreased with phylogenetic distance among host plants, one-tailed Mantel test was performed between Bray–Curtis similarity of the fungal community and phylogenetic distance among host plants in the R-package “ecodist.”

We also evaluated the network structural properties based on the species-level mangrove-epiphytic fungal and mangrove-endophytic fungal matrices. The network-level indices included specialization (Blüthgen et al., 2006), modularity (Beckett, 2016), weighted connectance (Tylianakis et al., 2007) and weighted nestedness metric based on the overlap and decreasing fill (WNODF) (Almeida-Neto and Ulrich, 2011). To enable comparisons across networks with different numbers of species and interactions, network metrics were standardized as z-scores. Z-score of a network metric was defined as z = (obs – exp)/sd.exp, where obs represented the observed value, and exp and sd.exp were the average value and the standard deviation of the 1,000 randomized network matrices (Ulrich et al., 2009). The 1,000 randomized network matrices were generated using the ‘swap’ method with marginal totals and connectance identical to observed network (Artzy-Randrup and Stone, 2005; Dormann et al., 2009).

Furthermore, we quantified network robustness by assessing the secondary extinctions of plant (fungal) communities to the primary random extinctions of fungi (plants) respectively. The curve of secondary extinction of the fungal (plant) community was fitted with an exponential regression model *y* ∼ 1 – *x*^a^ (Memmott et al., 2004; Burgos et al., 2007). In the model, *x* represented the proportion of target plant (fungal) species removed (primary extinction) and *y* represented the proportion of fungal (plant) species that still alive. The area below the curve (representing robustness, R) was used to quantify the tolerance of a system to the extinction of its component species (Memmott et al., 2004; Burgos et al., 2007). All network metrics were computed using the R-package “bipartite” (Dormann et al., 2009).

## Results

### Characterization of Illumina sequencing data

**Figure 1:**
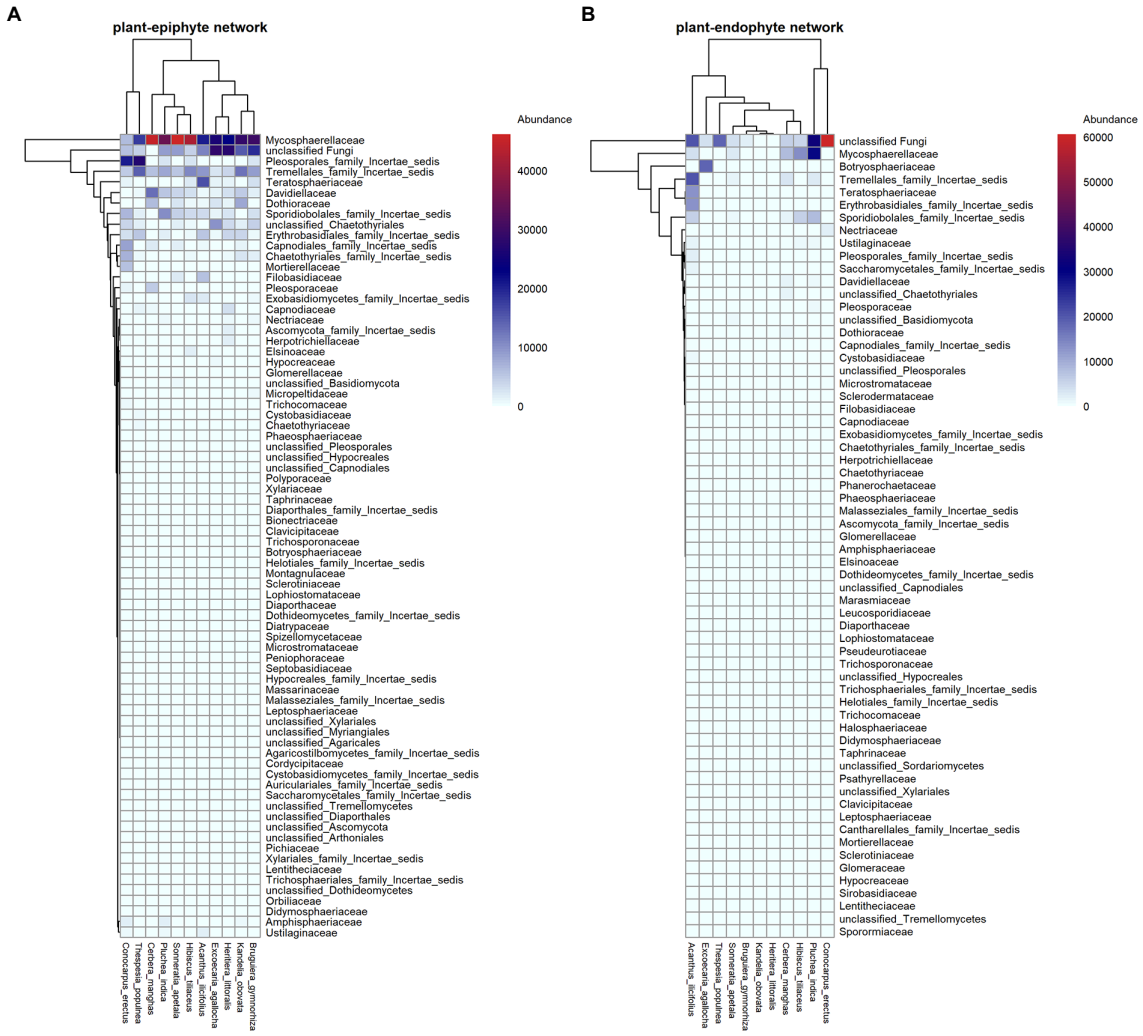
Interaction patterns of plant-endophytic fungi **(A)** and plant-epiphytic fungi **(B)**. In each panel, plant species and fungal families are shown in columns and rows, respectively. The relative abundance of each fungal family on host plant is filled in the heatmap.

Totally, after excluding 514 OTUs with <10 reads, we found 1,391 fungal OTUs on endophytes and epiphytes. The fungi included 528 Ascomycota, 275 Basidiomycota, 1 Cryptomycota, 1 Glomeromycota, 3 Zygomycota, and 493 unknown fungi at the phylum level. At class level, Dothideomycetes (64.77%), Tremellomycetes (14.65%), and Eurotiomycetes (5.78%) showed high abundance in epiphytic fungal communities, while Dothideomycetes (30.98%), Tremellomycetes (11.11%) and Microbotryomycetes (6.42%) showed high abundance in endophytic fungal communities. At family level, Mycosphaerellaceae (17.39%), and Davidiellaceae (5.97%) showed high abundance in endophytic fungal communities while Mycosphaerellaceae (37.32%) and Botryosphaeriaceae (4.36%) showed high abundance in epiphytic fungal communities (Figure 1).

### The richness of epiphytic and endophytic fungi

**Figure 2:**
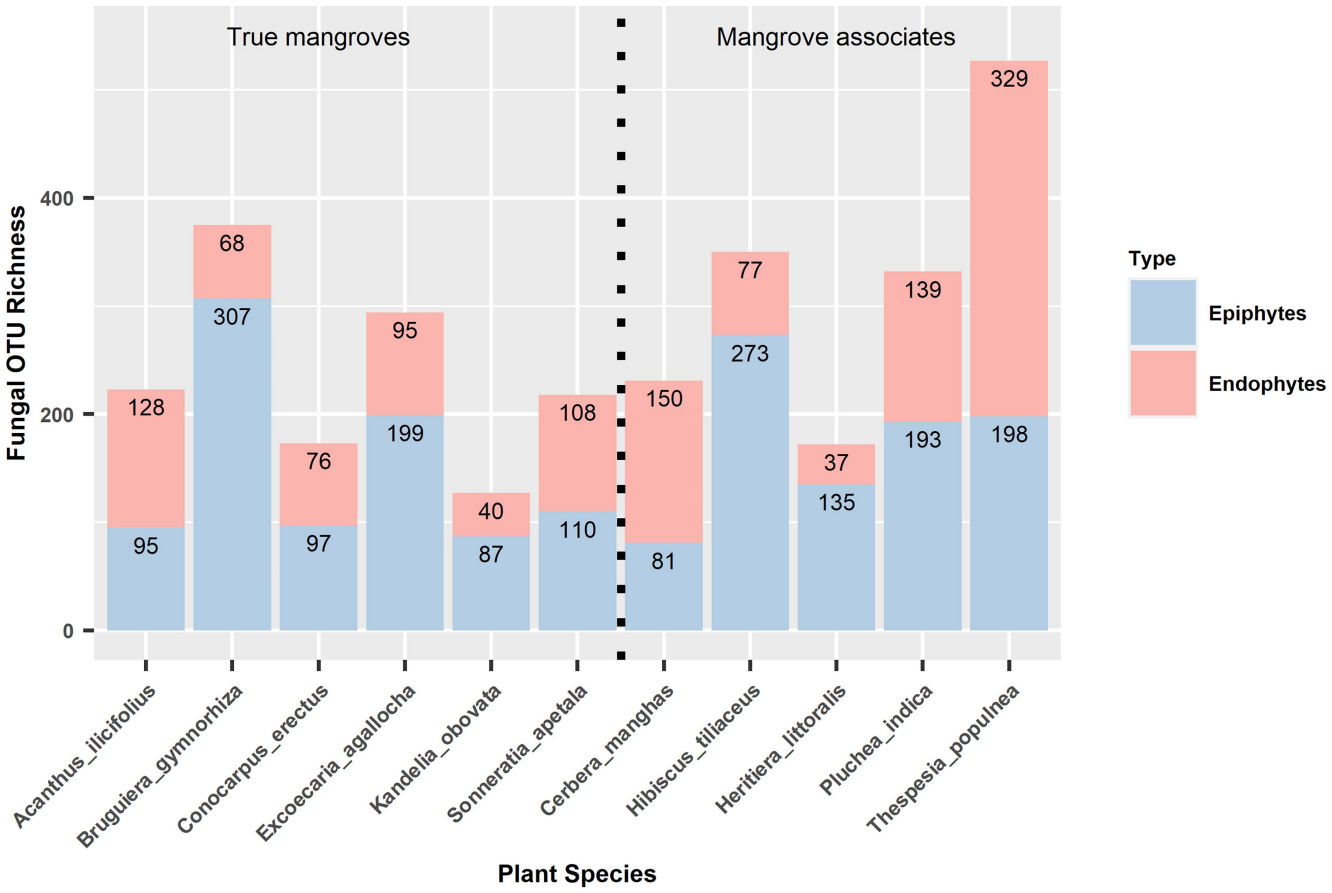
OTU richness of epiphytic and endophytic fungal communities in 11 host plant species. True mangroves and mangrove associates are showed on the left and right panels, separately.

Out of the 1,391 fungal OTUs, 596 (42.85% of the total OTUs) were exclusively epiphytic fungi, 600 (43.13%) were exclusively endophytic fungi, and 195 (14.02%) were shared between them. Across all 11 plant species, the average number of OTU richness of epiphytic fungal community per plant species was significantly higher than that of endophytic fungal community (paired *t*-test, *t* = 1.92, *df* = 10, *p* = 0.042). For instance, the OTU richness of epiphytic and endophytic fungi, respectively, was 273 and 77 in *Hibiscus tiliaceus*, 135 and 37 in *Heritiera littoralis*, 97 and 76 in *Conocarpus erectus*, 110 and 108 in *Sonneratia apetala*, 199 and 95 in *Excoecaria agallocha*, 87 and 40 in *Kandelia obovata*, 307 and 68 in *Bruguiera gymnorhiza*, 193 and 139 in *Pluchea indica* (Figure 2). No significant phylogenetic signals were detected in the richness for epiphytic fungi (Blomberg’s *K* = 0.248, *p* = 0.900; Pagel’s *lambda* = 6.611 × 10^−5^, *p* = 1) and endophytic fungi (Blomberg’s *K* = 0.206, *p* = 0.906; Pagel’s *lambda* = 6.611 × 10^−5^, *p* = 1) on each host plant species.

### Community composition of epiphytic and endophytic fungi

**Figure 3:**
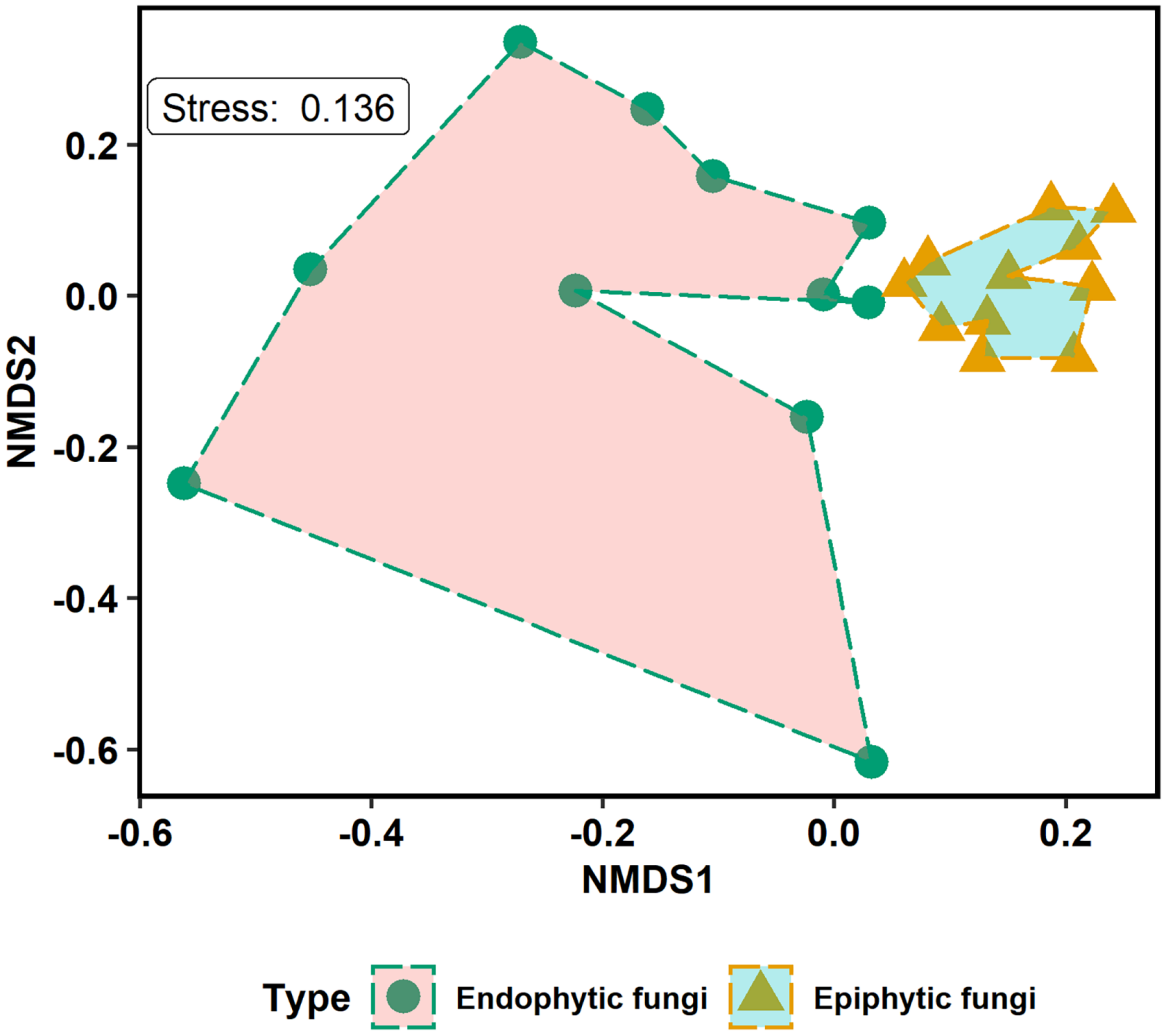
Non-metric multidimensional scaling (NMDS) ordination plot of the community composition of epiphytic and endophytic fungi in mangrove species. The endophytic and epiphytic fungi are grouped by fungal types. Kruskal’s stress value equals 0.136.

**Figure 4:**
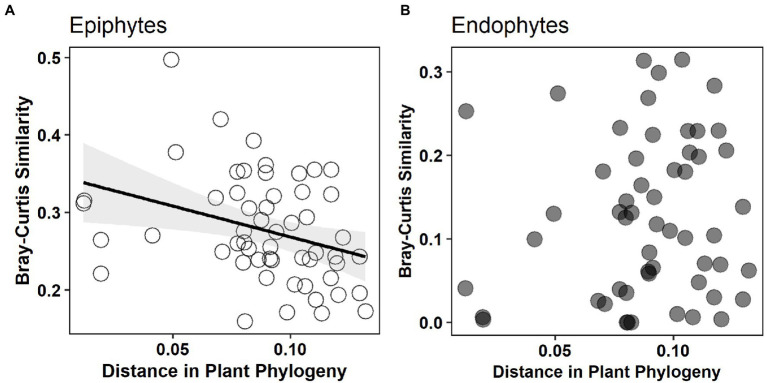
The relationship between Bray–Curtis similarity of epiphytic and endophytic fungal community and distance in plant phylogeny. Significant negative relation [**(A)** Mantel *r* = −0.332, *p* = 0.032] is shown for epiphytes but not for endophytes [**(B)** Mantel *r* = 0.099, *p* = 0.658].

The non-metric multidimensional scaling (NMDS) ordination analysis showed that the community composition of epiphytic and endophytic fungi was different (Figure 3). Analysis of similarities (ANOSIM) showed that there was a strong, statistically significant difference in epiphytic and endophytic fungal communities (ANOSIM statistic R: 0.447, *p* = 0.0001). One-tailed Mantel tests showed that there was a significant negative relation between community similarity for epiphytes and distance in plant phylogeny (Mantel *r* = −0.332, *p* = 0.032, see Figure 4A) but no such patterns for endophytes and distance in plant phylogeny (Mantel *r* = 0.099, *p* = 0.658, see Figure 4B).

### Network structures and robustness of plant–epiphytic fungi and plant–endophytic fungi

**Figure 5:**
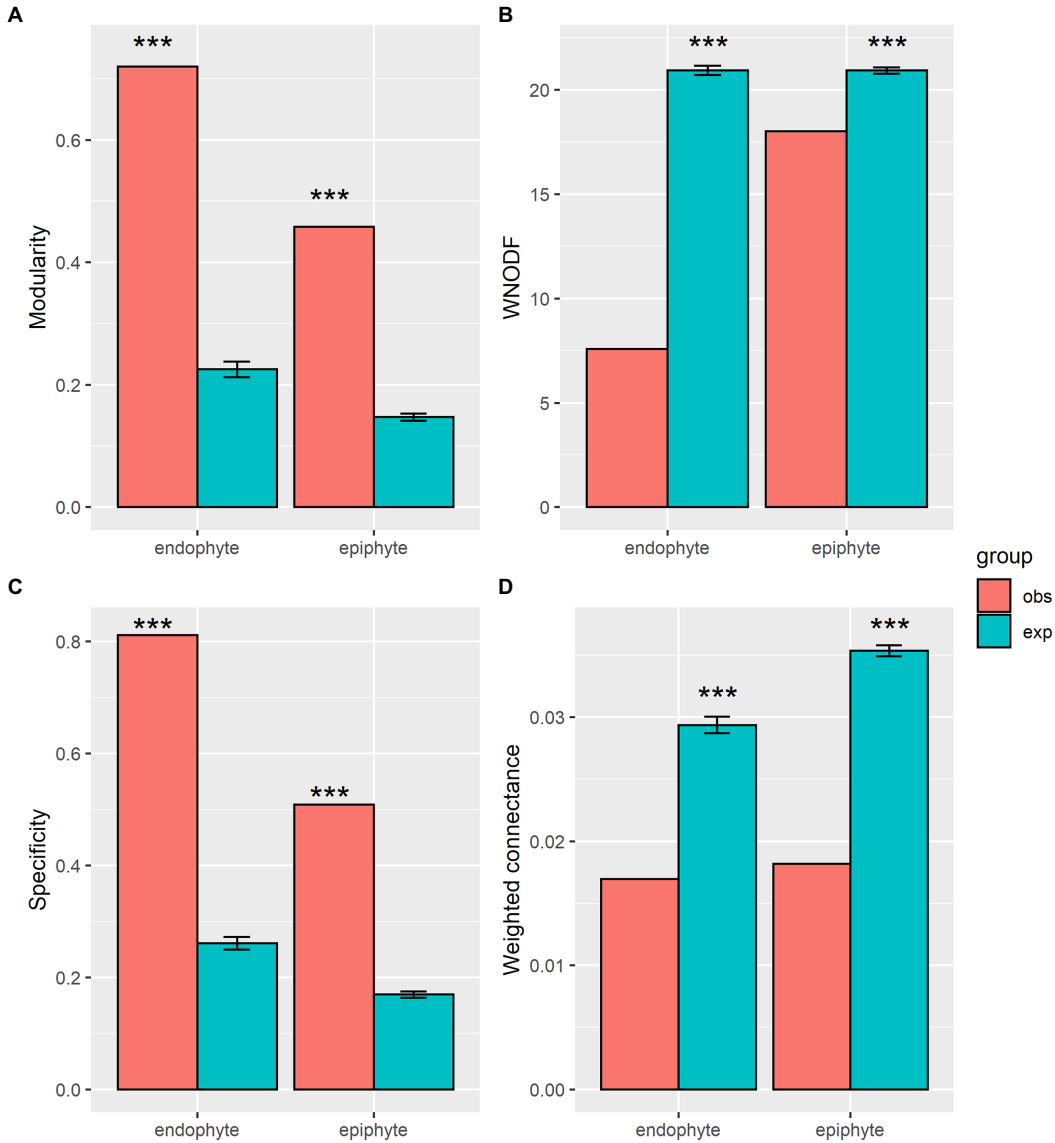
Network structural properties of plant-endophytic fungi and plant-epiphytic fungi. Network structures include modularity **(A)**, weighted nested [WNODF, **(B)**], specificity **(C)**, and weighted connectance **(D)**. Observation values (obs) of network metrics in our plant-fungal networks and expectation values (exp) and standard errors of that in 1,000 randomized networks, and significance level (* *p* < 0.05, ** *p* < 0.01, *** *p* < 0.001) are shown in the figure.

**Figure 6:**
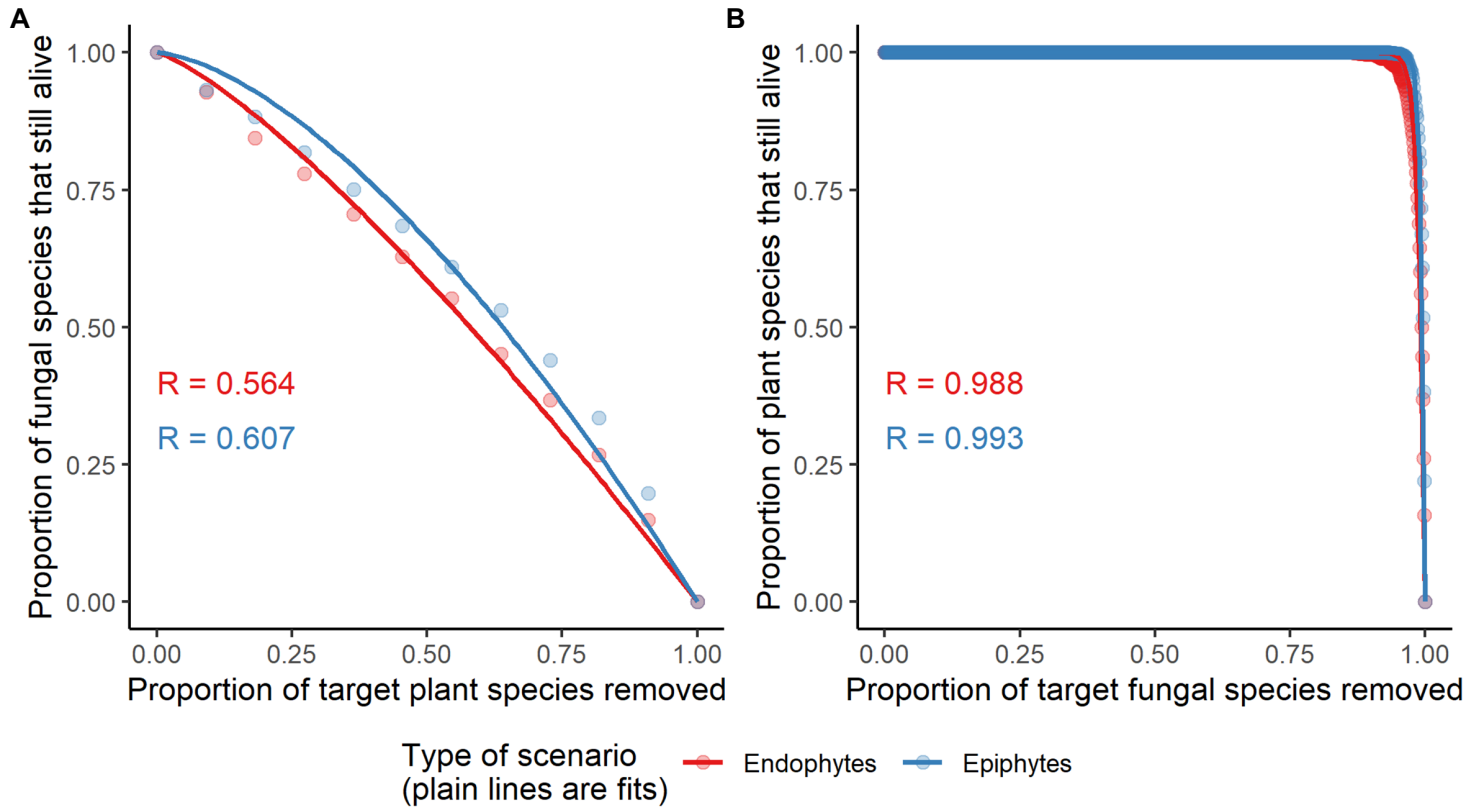
Network robustness of plant-endophytic fungi and plant-epiphytic fungi. The secondary extinctions of epiphytic and endophytic fungal species to random extinctions of host plant species are shown in panel **(A)**. The secondary extinctions of plant species to random extinctions of epiphytic and endophytic fungal species are shown in panel **(B)**. The area below the curve (representing network robustness, *R*) was used to quantify the tolerance of a system to the extinction of its component species. *R* values of network robustness are shown in each panel.

The observed values of specificity and modularity of both mangrove–epiphytic and –endophytic fungal networks were significantly higher than the expected values of randomized networks (Figures 5A,C), while the observed values of weighted connectance and weighted nestedness (WNODF) were significantly lower than the expectations of randomized networks (Figures 5B,D). Thus, mangrove–epiphytic and–endophytic fungal networks exhibited strong specialization and modularity but low connectance and anti-nestedness (i.e., lower nestedness than randomized networks) (see Figure 5). Moreover, mangrove–epiphytic fungal network had higher specialization (*z* = 59.90) and higher modularity (*z* = 52.15), lower connectance (*z* = −38.09), and lower anti-nestedness (*z* = −18.87) than mangrove–endophytic fungal network (specialization *z* = 47.78, modularity *z* = 39.23, connectance *z* = −18.56, nestedness *z* = −21.95). In addition, we found that epiphytic fungal communities were more robust than endophytic fungal communities when host plants were randomly removed. Meanwhile, host plants were more robust to random removal of epiphytic than endophytic fungi (Figure 6).

## Discussion

### The richness of epiphytic and endophytic fungal communities

In our mangrove ecosystem, species richness of epiphytic fungal community per plant species was higher than that of endophytic fungal community. Lower richness for endophytes than epiphytes may be explained by the fact that endophytes live in a more constant environment sheltered from environmental conditions by plant tissues (Mercado-Blanco, 2015), whereas epiphytes may need to cope with complicated seaside environments of high salinity like other microbes living in mangrove forests (Ceccon et al., 2019). Besides, such difference in fungal richness may be attributed to that a few fungi can enter the plant tissues through natural openings such as stomata and hydathodes athough many fungi arrive on the surfaces of leaf (Vorholt, 2012). Similarly, prior studies in other ecosystems also found that phyllosphere endophytic and epiphytic fungal communities were different, with epiphytic communities being richer and more abundant on live trees (Gomes et al., 2018) and *Eucalyptus citriodora* Hook (Kharwar et al., 2010). Contrary to this study, for leaf fungi on true mangroves, Yao et al. (2019) found lower richness of epiphytic than endophytic fungal community. Such inconsistent patterns may be explained by different mangrove plants investigated at the two study sites, with evidence showing that selective pressures exerted by the plant upon phyllosphere microbes vary from one host plant to the other (Vacher et al., 2016). Besides, a low richness of epiphytes associated with mangroves at low latitude site (Yao et al., 2019) may be attributed to that strong desiccation and UV radiations can exert selection pressure on colonization and reproduction of epiphytes on the leaf surfaces (Lee et al., 2019).

### Community composition of epiphytic and endophytic fungi

Phyllosphere fungal communities were majorly dominated by Ascomycota phylum, followed by Basidiomycota in our mangrove ecosystem, in agreement with previous findings in mangroves (Chi et al., 2019; Devadatha et al., 2021). Low richness of Basidomycota living in the phylllosphere may be explained by dispersal limitation due to that most Basidiomycota spores dispersed over short distances from sporocarps (Galante et al., 2011). At the class level, Dothideomycetes and Tremellomycetes were most dominant in both epiphytic and endophytic leaf fungal communities in our mangrove forests. Similarly, the pattern that Dothideomycetes was a predominant fungal class was also reported in other studies (Dong et al., 2021; Zhu et al., 2021). This may be explained by that Dothidiomycetes is the largest class of kingdom fungi, comprising most ecologically diverse of fungi (Kirk et al., 2008).

The community composition of epiphytic and endophytic fungi differed significantly in our mangrove ecosystem, as reported in the mangrove and other woody ecosystems (Kembel and Mueller, 2014; Gomes et al., 2018; Yao et al., 2019). As well, the predominant composition of epiphytic and endophytic fungal communities remained different on the three true mangroves shared between this local and a previous mangrove ecosystem (Supplementary Table S2). First, this may be explained by that different external environmental factors would shape differentiated fungal communities of epiphytes (e.g., season and wind speed) and endophytes (e.g., season and rainfall) (Osono, 2017; Gomes et al., 2018). Second, this may be attributed to the variations in leaf functional traits (e.g., nutrients, leaf physical and chemical properties) which have been broadly reported to affect community assembly of leaf endophytic fungi (Sun et al., 2014; González-Teuber et al., 2020; Tellez et al., 2022) and epiphytic fungi (Inácio et al., 2002; Kembel and Mueller, 2014; Tang et al., 2021; Li et al., 2022).

In addition, community of endophytic fungi has been reported to experience stronger effect from host plant identity than that of epiphytic fungi in a mangrove ecosystem (Yao et al., 2019). However, we found that epiphytic fungal communities were significantly constrained by plant phylogeny but no such constraint effects were observed for the endophytic fungal communities (Figure 4). This pattern is possibly due to that epiphytic fungal communities are majorly shaped and filtered by phylogenetically conservative plant functional traits whereas endophytic fungal communities may be majorly selected by phylogenetically dispersion plant functional traits (e.g., experienced strong natural selection in the evolutionary history). Some evidence showed that from epiphytes to endophytes, host selection pressure sequentially increased, along with the strongest host selection pressure in the leaf endosphere (Xiong et al., 2021).

### Network structures and robustness of plant–epiphytic and plant–Endophytic fungi

Our mangrove–epiphytic and–endophytic fungal networks exhibited strong specialization and modularity, but low connectance and anti-nestedness, in accordance with network patterns of true mangrove–epiphytic and –endophytic fungus (Yao et al., 2020). Similarly, highly specialized and modular but anti-nestedness structures were generally found in the belowground plant–ectomycorrhizal fungal networks (Bahram et al., 2014; Jacquemyn et al., 2014) and ericaceous plant–root fungal networks (Toju et al., 2016). Furthermore, our mangrove–epiphytic fungal network showed stronger specialization and modularity and lower connectance compared to mangrove–endophytic fungal network, contrary to previous findings (Yao et al., 2020). This indicates that additional investigation concerning phyllosphere fungi on mangrove associates can probably improve our knowledge about community assembly of epiphytic and endophytic fungi in mangrove ecosystems. For instance, higher specialization of mangrove associates–epiphytic than –endophytic fungal network (Supplementary Table S3) might make large contribution to the observed higher specialization in our mangrove–epiphytic than –endophytic fungal network.

The stronger anti-nestedness was observed in our mangrove–endophytic fungal network than mangrove–epiphytic fungal network. This suggests that specialized mangrove plants may prefer to interact with specialists over generalists among endophytic fungi than epiphytic fungi (Chagnon et al., 2016). This can be attributed to strong selection pressure of host in choosing its phyllosphere fungi as supported by the constraint effect of host phylogeny on epiphytic fungal community (Figure 4). However, high specialization and modularity may result from a strong host partner selectivity of phyllosphere fungi. The higher specialization and modularity in the mangrove–epiphytic fungal network may be explained by stronger host specificity and niche differentiation exhibited by epiphytic than endophytic fungal species (Supplementary Table S4). Such higher specialization and modularity may further promote higher network robustness of mangrove–epiphytic fungi than mangrove–endophytic fungi (Figure 6). In addition, infection and colonization of phyllosphere fungi also depend on phyllosphere microenvironment such as sunlight intensity, moisture and leaf physical and chemical properties (Osono, 2014; González-Teuber et al., 2020), showing that local unmeasured microenvironment and host plant properties may cofound network structures.

Network structures may be affected by the sampling and network matrix properties. For instance, a lower number of sampled host species may lead to weaker modularity (Põlme et al., 2018) and anti-nestedness (Bahram et al., 2014) within the plant–fungal networks. The anti-nestedness property in our mangrove–phyllosphere fungal networks may be affected by a low number of host plants (six true mangrove species and five mangrove associates) but modularity seems to be not. Further, high nestedness and modularity may be associated with increasing connectance (Fortuna et al., 2010; Põlme et al., 2018). For instance, exclusion of rare associations (doubletons but not singletons) has been confirmed to enhance nestedness (Põlme et al., 2018). In our study, anti-nestedness in mangrove–phyllosphere fungal networks may be partially attributed to low connectance. Such low connectance may be a result of rare associations which are more likely captured by high throughput sequencing technology.

## Conclusion

By assessing the differences in community and network structures of phyllosphere epiphytic and endophytic fungi in a subtropical mangrove ecosystem, we uncover the mechanisms of fungal community assembly. Particularly, we find that phyllosphere epiphytic fungal communities are constrained by phylogeny of host plant, revealing the important role of host plant in shaping microbial community. As well, mangrove–epiphytic and –endophytic fungal networks are not randomly assembled. Compared to endophytic fungal network, epiphytic fungal network exhibits stronger specialization, modularity and robustness, similar to the network patterns between epiphytic and endophytic bacterial networks (Yao et al., 2020). This indicates that host preference and niche partitioning might contribute to that epiphytic fungal communities are more resistant to external environmental perturbations and species extinctions than endophytic fungal communities. These findings will improve our understanding of the community structure and dynamics of the phyllosphere microorganisms in mangrove ecosystems.

## Data availability statement

All raw sequences presented in the study are deposited in the SRA database of the National Center for Biotechnology Information, accession number PRJNA938903 (https://www.ncbi.nlm.nih.gov/bioproject/938903).

## Author contributions

CZ designed the research, analyzed the data, and lead the writing. YL collected the samples and analyzed the data. ZW, WL, YZ, and CC revised and improved the writing. All authors contributed to the article and approved the submitted version.

## Funding

This research was financially supported by the Doctoral Startup Project of Zunyi Medical University (F-ZH-002), the Science and Technology Program of Guizhou Province, China (QKHJC- ZK [2021] 096), and the National Natural Science Foundation of China [grant numbers 32101281].

## Conflict of interest

YL is employed by Goldpac limited.

The remaining authors declare that the research was conducted in the absence of any commercial or financial relationships that could be construed as a potential conflict of interest.

## Publisher’s note

All claims expressed in this article are solely those of the authors and do not necessarily represent those of their affiliated organizations, or those of the publisher, the editors and the reviewers. Any product that may be evaluated in this article, or claim that may be made by its manufacturer, is not guaranteed or endorsed by the publisher.
