# Supplementary material for: Community assembly and network structure of epiphytic and endophytic phyllosphere fungi in a subtropical mangrove ecosystem
Source: Front Microbiol. 2023 Mar 17;14:1147285. doi: 10.3389/fmicb.2023.1147285 (PMC10064055; doi:10.3389/fmicb.2023.1147285)
Supplement: Supplementary file 1 [file Data_Sheet_1.docx]

**Supplementary Material**

**Figure S1.** The phylogenetic tree of six true mangroves and five mangrove associates in the mangrove nature reserve of Qi’ao island, South China. The percentage of replicate trees in which the associated taxa cluster together in the bootstrap test (1000 replicates) are shown next to the branches.

**Table S1.** The details about *rbcL*a barcode sequences deposited in the National Center for Biotechnology Information (NCBI). *RbcL*a sequences are used to construct phylogeny of host plants (Figure S1).

| DNA barcode | plant species | Accession no. | References |
| --- | --- | --- | --- |
| *rbcL*a | *Hibiscus tiliaceus* | GU135284.1 | Abbott et al., unpublished |
| *rbcL*a | *Cerbera manghas* | OK634680.1 | Mao et al., 2021 |
| *rbcL*a | *Excoecaria agallocha* | OK634680.1 | Mao et al., 2021 |
| *rbcL*a | *Pluchea indica* | OK635050.1 | Mao et al., 2021 |
| *rbcL*a | *Acanthus ilicifolius* | OK634431.1 | Mao et al., 2021 |
| *rbcL*a | *Bruguiera gymnorhiza* | OK634598.1 | Mao et al., 2021 |
| *rbcL*a | *Kandelia obovata* | OK634929.1 | Mao et al., 2021 |
| *rbcL*a | *Sonneratia apetala* | OK635192.1 | Mao et al., 2021 |
| *rbcL*a | *Heritiera littoralis* | OK634837.1 | Mao et al., 2021 |
| *rbcL*a | *Thespesia populnea* | OK635252.1 | Mao et al., 2021 |
| *rbcL*a | *Conocarpus erectus* | OK634748.1 | Mao et al., 2021 |

**Table S2.** A comparison of dominant community composition of leaf epiphytic and endophytic fungi on three same mangrove species (*Excoecaria agallocha*, *Bruguiera gymnorhiza* and *Kandelia obovate*) between this study and a previous study (Yao et al., 2019). In this study, the sampling site of mangroves located at Zhuhai, Guangdong, China, while the sampling site of a previous study located at Zhanjiang, Guangdong, China (Yao et al., 2019). The top three abundant classes of epiphytic and endophytic fungi identified in the two studies are illustrated. The dominant fungal classes were denoted with a percentage value of fungal abundance in our study site.

|  | Dominant fungi at Zhanjiang | | Dominant fungi at Zhuhai | |
| --- | --- | --- | --- | --- |
|  | Epiphytes | Endophytes | Epiphytes | Endophytes |
| *Excoecaria*  *agalloch* | Dothideomycetes | Dothideomycetes | Dothideomycetes  (48.62%) | Dothideomycetes  (50.24%) |
|  | Exobasidiomycetes | Exobasidiomycetes | Eurotiomycetes (39.69%) | Eurotiomycetes  (1.54%) |
|  | Tremellomycetes | Tremellomycetes | Tremellomycetes  (5.88%) | Tremellomycetes  (0.27%) |
| *Kandelia obovate* | Dothideomycetes | Dothideomycetes | Dothideomycetes  (57.14%) | Dothideomycetes  (9.32%) |
|  | Tremellomycetes | Tremellomycetes | Tremellomycetes  (20.94%) | Sordariomycetes  (6.40%) |
|  | Microbotryomycetes | Microbotryomycetes | Eurotiomycetes  (10.40%) | Tremellomycetes  (2.38%) |
| *Bruguiera gymnorhiza* | Dothideomycetes | Dothideomycetes | Dothideomycetes  (54.09%) | Dothideomycetes  (4.08%) |
|  | Tremellomycetes | Tremellomycetes | Eurotiomycetes  (19.52%) | Eurotiomycetes  (1.68%) |
|  | Microbotryomycetes | Microbotryomycetes | Tremellomycetes  (12.80%) | Sordariomycetes  (1.29%) |

**Table S3.** Specialization of true mangrove–epiphytic and –endophytic fungal and mangrove associate–epiphytic and –endophytic fungal networks. The z-transformed specialization of each subnetwork is shown in the table.

|  | Epiphytic fungi | Endophytic fungi |
| --- | --- | --- |
| True mangroves | 25.00 | 22.17 |
| Mangrove associates | 25.75 | 14.00 |

**Table S4.** Specialization of epiphytic and endophytic fungi and their host plants. The species-level specialization index is quantified using d' index developed by Blüthgen et al. (2006). The specialization of each trophic level is quantified using mean values of d’ and its z-score.

|  | Observed mean d' | Z-score of observed mean d' |
| --- | --- | --- |
| Epiphytic fungi | 0.24 | 63.22 |
| Epiphytic fungal hosts | 0.48 | 59.88 |
| Endophytic fungi | 0.30 | 34.48 |
| Endophytic fungal hosts | 0.76 | 27.68 |

**References**

Abbott, J.R., Neubig, K.M., Whitten, W.M. and Williams, N.H. DNA barcoding the flora of Florida: invasive Species. Unpublised.

Blüthgen, N., Menzel, F., and Blüthgen, N. (2006). Measuring specialization in species interaction networks. *BMC Ecology* 6, 9. doi:10.1186/1472-6785-6-9.

Mao, X., Xie, W., Li, X., Shi, S., and Guo, Z. (2021). Establishing community-wide DNA barcode references for conserving mangrove forests in China. *BMC Plant Biology* 21, 571. doi:10.1186/s12870-021-03349-z.

Yao, H., Sun, X., He, C., Maitra, P., Li, X., and Guo, L. (2019). Phyllosphere epiphytic and endophytic fungal community and network structures differ in a tropical mangrove ecosystem. *Microbiome* 7, 57. doi:10.1186/s40168-019-0671-0.
